# Fine Mapping of *qRC10-2*, a Quantitative Trait Locus for Cold Tolerance of Rice Roots at Seedling and Mature Stages

**DOI:** 10.1371/journal.pone.0096046

**Published:** 2014-05-01

**Authors:** Ning Xiao, Wei-nan Huang, Xiao-xiang Zhang, Yong Gao, Ai-hong Li, Yi Dai, Ling Yu, Guang-qing Liu, Cun-hong Pan, Yu-hong Li, Zheng-yuan Dai, Jian-min Chen

**Affiliations:** 1 College of Bioscience and Biotechnology and Jiangsu Key Laboratory of Crop Genetics and Physiology, Yangzhou University, Yangzhou, Jiangsu Province, China; 2 Lixiahe Agricultural Research Institute of Jiangsu Province, National Rice Industry Technology System of Yangzhou Comprehensive Experimental Station, Yangzhou, Jiangsu Province, China; China Agricultural University, China

## Abstract

Cold stress causes various injuries to rice seedlings in low-temperature and high-altitude areas and is therefore an important factor affecting rice production in such areas. In this study, root conductivity (RC) was used as an indicator to map quantitative trait loci (QTLs) of cold tolerance in *Oryza rufipogon* Griff., Dongxiang wild rice (DX), at its two-leaf stage. The correlation coefficients between RC and the plant survival rate (PSR) at the seedling and maturity stages were –0.85 and –0.9 (*P* = 0.01), respectively, indicating that RC is a reliable index for evaluating cold tolerance of rice. A preliminary mapping group was constructed from 151 BC_2_F_1_ plants using DX as a cold-tolerant donor and the *indica* variety Nanjing 11 (NJ) as a recurrent parent. A total of 113 codominant simple-sequence repeat (SSR) markers were developed, with a parental polymorphism of 17.3%. Two cold-tolerant QTLs, named *qRC10-1* and *qRC10-2* were detected on chromosome 10 by composite interval mapping. *qRC10-1* (LOD = 3.1, *RM171*-*RM1108*) was mapped at 148.3 cM, and *qRC10-2* (LOD = 6.1, *RM25570*-*RM304*) was mapped at 163.3 cM, which accounted for 9.4% and 32.1% of phenotypic variances, respectively. To fine map the major locus *qRC10-2*, NJ was crossed with a BC_4_F_2_ plant (L188-3), which only carried the QTL *qRC10-2*, to construct a large BC_5_F_2_ fine-mapping population with 13,324 progenies. Forty-five molecular markers were designed to evenly cover *qRC10-2*, and 10 markers showed polymorphisms between DX and NJ. As a result, *qRC10-2* was delimited to a 48.5-kb region between markers *qc45* and *qc48*. In this region, *Os10g0489500* and *Os10g0490100* exhibited different expression patterns between DX and NJ. Our results provide a basis for identifying the gene(s) underlying *qRC10-2*, and the markers developed here may be used to improve low-temperature tolerance of rice seedling and maturity stages via marker-assisted selection (MAS).

**Key Message:**

With root electrical conductivity used as a cold-tolerance index, the quantitative trait locus *qRC10-2* was fine mapped to a 48.5-kb candidate region, and *Os10g0489500* and *Os10g0490100* were identified as differently expressed genes for *qRC10-2*.

## Introduction

Rice (*Oryza sativa* L.) is one of the three major food crops in the world and feeds about 21% of the global population in terms of per capita energy [1]. Cold stress is a common problem for rice cultivation and is a crucial factor affecting global food production [2]. A primary objective for rice breeding in low-temperature and high-altitude areas is to improve cold tolerance of rice cultivars. Therefore, mining cold-tolerant germplasms and mapping related genes would provide an opportunity to improve rice yield through marker-assisted selection (MAS) [3]–[5]. Recently, several quantitative trait loci (QTLs, e.g., *Ctb-1*, *Ctb-2*, *qCTB3*, *qCTB7*, and *qCTB8*) related to cold-induced rice damages to aboveground organs–including seedling mortality and growth, leaf wilting, and necrotic yellowing–were mapped [3], [6]–[11]. To date, however, only *Ctb-1*, which improves the seed setting rate by enhancing spikelet fertility, has been isolated and cloned and has been proven to be involved primarily in the ubiquitin-proteasome pathway [8]. QTLs contributing to cold tolerance of seedlings are located on several rice chromosomes, accounting for 4–49.3% of phenotypic variations [4], [12]–[22]. In addition, certain chromosomes also bear germination cold-tolerant QTLs [23], [24]. Cold tolerance of rice is controlled by multiple genes, and different cold-tolerance-related mechanisms take effect during the growth period of rice.

Nevertheless, cold-tolerant QTLs have not been reported in rice roots, an important underground organ that protects rice from cold stress. At low temperatures, the integrity of plant cellular membranes can be compromised by the accumulation of peroxide and oxide radicals, which increasing membrane permeability [25]–[27]. Rice roots are more sensitive to low temperature than leaves, and cold resistance at the seedling stage can be directly determined by the contribution of roots to cold resistance [28]. Therefore, root conductivity (RC) tests have been used to determine the degree of cold-induced injuries in wheat [29]. However, cold-tolerant QTLs have not been reported in rice roots based on the RC test.

Dongxiang wild rice (DX), a Chinese common wild rice (*Oryza rufipogon* Griff., AA genome) grown in Jiangxi Province (116°36′ E, 28°14′ N), is the northernmost wild rice known in the world [30]. It possesses extremely high tolerance to cold stress and is able to safely survive winter temperatures as low as –12.8°C [31]. In our present study, we reported fine mapping of a major QTL, *qRC10-2*, related to root cold tolerance using DX as a cold-tolerant donor and the cold-sensitive *indica* variety Nanjing 11 (NJ) as a recurrent parent. We also evaluated the effects of *qRC10-2* at the seedling and maturity stages. The analysis of the QTL derived from the wild rice will provide novel alleles for improving rice cold tolerance by MAS and also genetic materials for understanding the molecular mechanism of cold tolerance in rice.

## Materials and Methods

### Plant Materials

**Figure 1:**
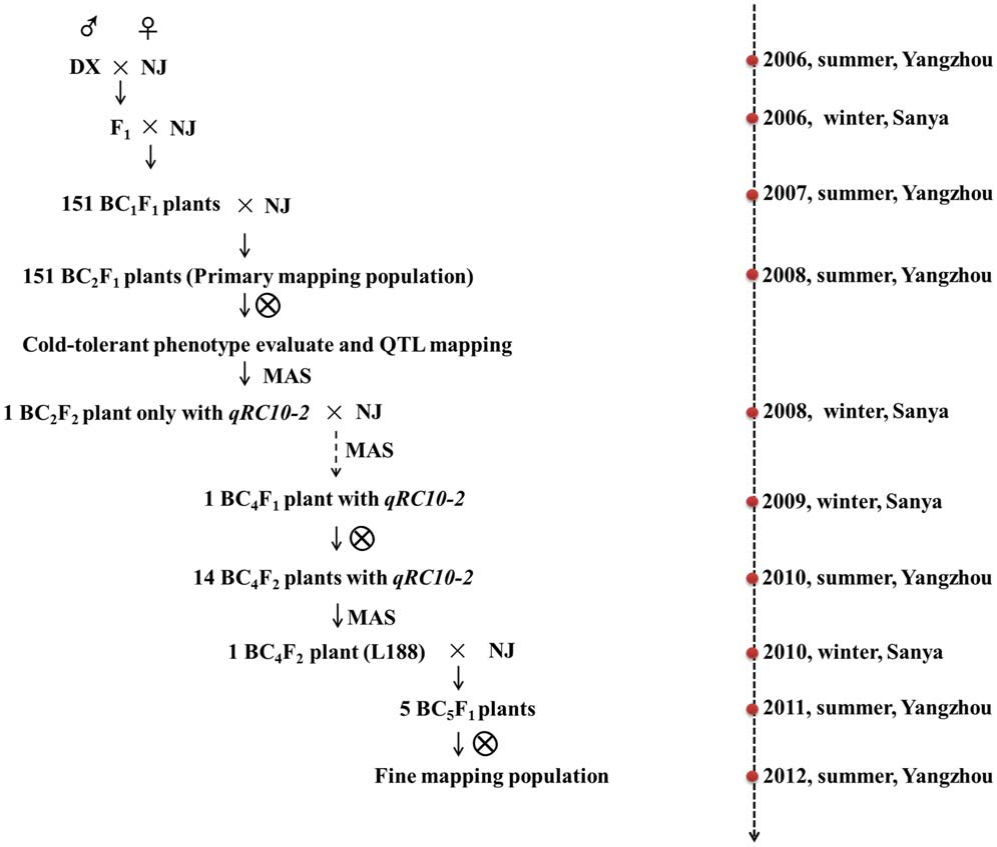
Process of constructing the mapping population.

The cold-tolerant variety DX was used as a donor parent to cross with the cold-sensitive NJ in the summer of 2006 (Fig. 1). Then, a primary mapping population was constructed by employing the advanced backcross approach described by Thomson et al. [32]. The F_1_ progeny was first backcrossed to NJ to produce 151 BC_1_F_1_ plants, which were then grown in a greenhouse, and subsequently each BC_1_F_1_ plant was individually backcrossed to the recurrent parent until BC_2_F_1_ was obtained. No cold phenotype was selected for RC during population development because phenotype evaluation is destructive and thus cannot be conducted on individual plants. All BC_2_F_2_ seeds from 151 BC_2_F_1_ plants were individually harvested, and the resulting population of 151 individual BC_2_F_2_ families was used for RC phenotypic characterization in September of 2008.

To eliminate the effect of *qRC10-1*, a single BC_2_F_2_ plant that contained the DX-introgressed segment at the locus *qRC10-2* was selected for continuous backcrossing with NJ followed by MAS using the markers *RM25570* and *RM304*. This yielded 14 BC_4_F_2_ backcross inbred lines (BILs). Later, 150 simple-sequence repeat (SSR) markers covering the whole genome were used to determine the genetic background recovery rates, which varied from 89.33% to 94.67% (Fig. S1). Among these BILs, L188-3 (hereafter, L188) had a background recovery rate of 94.67%, which was found to have low RC. A large BC_5_F_2_ fine-mapping population of 13,324 progenies was constructed by crossing NJ with L188 in the summer of 2012. Then, molecular markers were used to detect recombinant events covering *qRC10-2.* All needed recombinants were separately harvested and evaluated for RC, and the BC_5_F_4_ seeds from the BC_5_F_3_ lines were harvested to calculate the plant survival rate (PSR) at the seedling and maturity stages. All plants were grown in the Yangzhou Wantou experimental fields at the Lixiahe Agricultural Research Institute of Jiangsu Province (119°42′ E, 32°39′ N) in summer and in Sanya of Hainan Province (110°02′ E, 18°48′ N) in winter.

### Evaluation of Cold Tolerance

The RC phenotypes of the BC_2_F_1_ and BC_5_F_2_ plants were measured as the average RC of the derived BC_2_F_2_ and BC_5_F_3_ plants across three replicates, respectively. Twenty seeds were randomly selected and placed in an illuminated incubator for growth to day 9. The seedlings were then given simulated diurnal alternating illumination treatment: 12 h dark at 4±1°C versus 12 h with 25,000 Lx of illumination at 4±1°C with relative humidity of 75–85%. After 48 h treatment, the 2-cm taproot section was immediately cut from each plant, and placed into an Eppendorf centrifuge tube with 2 mL deionized H_2_O (conductivity of 1.5 µs/cm). After soaking for 1 h, the RC of each plant was measured with a conductivity meter (Mettler Toledo 326) and data were adjusted for RC of deionized H_2_O.

Approximately 100 BC_5_F_4_ seeds were selected from each BC_5_F_3_ line with high or low RC to evaluate the PSR using the criteria of RC<10 and RC≥10 for cold tolerance and cold sensitivity, respectively. At day 9, the seedlings were cold-treated as described above and then returned to constant 28°C for recovery under illumination at 25,000 Lx for 72 h. The plants were divided into a sensitive group (with completely-withered leaves) and a tolerant group (normal growth). PSR was estimated as the proportion of the plants showing a tolerant phenotype to all the plants by percentage (0–100%). The PSR of each BC_5_F_3_ line was measured in three replicates.

Root cold tolerance at maturity was evaluated as follows: The BC_5_F_4_ seedlings obtained from the BC_5_F_3_ lines were planted in the field on May 10, 2013. Uniform seedlings were then transplanted into a hydroponic greenhouse on June 10 at Yangzhou University. The hydroponic pool was 880 cm long, 130 cm wide, and 50 cm deep. The cement anchoring the rice plants was 135 cm long, 16.7 cm wide, and 2.5 cm thick, and each had a plate of 14 holes that were 4.0 cm in diameter and away from each other by 10 cm. A single seedling was planted in each hole, which was fixed by cork and sponges. Each group (low RC vs. high RC) contained 126 plants in three replicates. The hydroponic solution (pH 5.8) contained 10 p.p.m. of nitrogen (NH_4_NO_3_), phosphorus (NaH_2_PO_4_), potassium (K_2_SO_4_), calcium (CaCl_2_), and magnesium (MgSO_4_) each, 2 p.p.m. of iron (Fe(III)-EDTA), and 0.5 p.p.m. of manganese (MnCl_2_), and the solution was changed every other day. An oxygen pump was used to provide a steady flow of hydroponic nutrients, which maintained the nutrient concentrations and pH conditions in the pool during plant growth from seedling to maturity. These concentrations were elevated to twice the starting concentrations 60 days later. After 15-day post-heading, the cold hydroponic solution at 5±1°C was circulated around the roots for 24 h. After recovery at 28°C for two days, the PSR was measured.

### Linkage Mapping and QTL Analysis

A genetic linkage map was constructed with Mapmaker/Exp 3.0 [33]. The segregation data of each marker were statistically analyzed to measure the segregation distortion from Mendelian expectations. Table S1 lists the SSR markers. QTL analysis was conducted by composite interval mapping using QTL Cartographer 2.5 [34]–[36]. The logarithm of odds (LOD) threshold of 2.5 was used to identify QTLs while avoiding false positives.

### DNA Extraction and Molecular Marker Analysis

DNA was extracted from leaves following the cetyltrimethylammonium bromide (CTAB) method [37]. All leaves were stored at –80°C before extraction. Additional SSR markers between *RM25570* and *RM304* were chosen from the Gramene database (http://www.gramene.org). The sequences of *indica* cv. 93-11 and *japonica* cv. Nipponbare were downloaded from IRGSP1.0 (http://rapdb.dna.affrc.go.jp/) and used to design insertion/deletion (indel) markers and real-time polymerase chain reaction (PCR) markers using Premier 5.0 (Tables S2 and S3).

PCR and electrophoresis were performed following the method of Panaud et al. [38]. PCR was performed in a 20 µL volume containing 20 ng of template DNA, 0.15 µL of 10 mM dNTPs, 2 U of *Taq* DNA polymerase, 2 µL of 10×PCR buffer (50 mM KCl, 10 mM Tris-HCl pH 8.3, 1.5 mM MgCl_2_, and 0.01% gelatin), and 1.5 µL of 2 µM forward and reverse primers each. The cycling conditions were 5 min at 94°C, followed by 35 cycles of 94°C for 1 min, 55°C for 1 min, and 72°C for 1 min, with a final extension at 72°C for 10 min. The PCR products were subjected to electrophoresis on 6% denaturing polyacrylamide gels, which were then stained with silver [39].

### Data Analysis

Differences between parents and BILs for cold-related measurement were compared via *t*-test using SPSS 11.0 for Windows (SPSS Inc., 2002). The correlation coefficient between RC and the PSR was calculated using the SAS software (SAS Institute, 2000).

### Gene Expression Analysis

At the two-leaf stage after cold treatment for 0, 6, 12, or 24 h, the total RNA was extracted from 100 mg roots and 100 mg leaves using the RNeasy extraction kit (Invitrogen, Carlsbad, CA, USA), and any remaining genomic DNA was removed by *DNase*I treatment. Total RNA (3 µg) was used as a template for cDNA synthesis using M-MLV transcriptase (TaKaRa Biotechnology, Dalian, China) with oligo (dT)_18_ primer. On an ABI PRISM 7500 system (Applied Biosystems, Foster City, CA, USA), real-time PCR was performed in a 20 µL volume containing 2 µL of first-strand cDNA, 10 µL of 2×SYBR Premix Ex Taq (TaKaRa, Shiga, Japan), 0.4 µL of 50×ROX reference dye, and 2 µL of each primer (2.5 µM). The used amplification protocol was 95°C for 30 s, followed by 40 cycles of 95°C for 5 s and 60°C for 30 s. Rice *Actin 1* was used as an internal control, and each experiment was repeated at least three times.

## Results

### RC is a Reliable Indicator of Cold Resistance in Rice Seedlings

**Figure 2:**
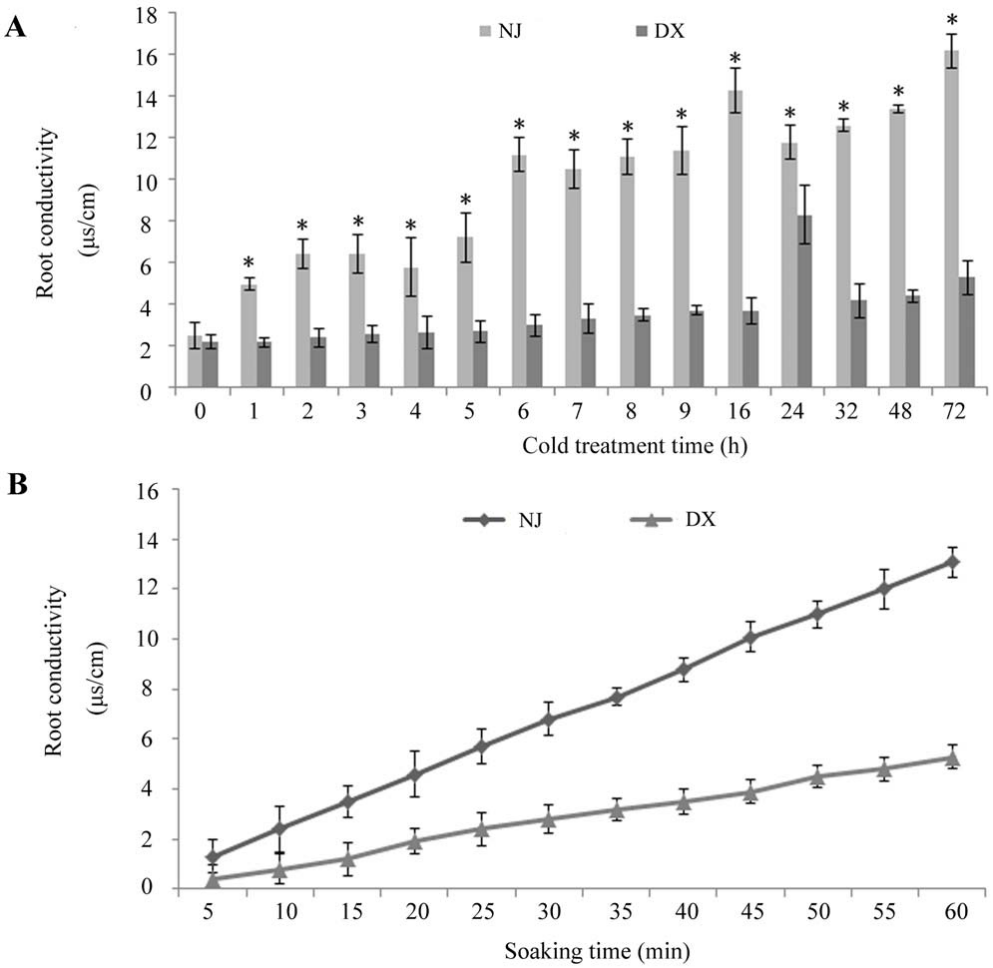
RC of DX and NJ as the function of the time exposed to cold and soaking. A. RC measured as a function of cold treatment duration. B. RC measured as a function of soaking time. An asterisk (*) indicates that the RC between NJ and DX was significantly different (*P*<0.01).

RC was significantly different for DX and NJ at day 8 (*P* = 0.01, Table 1). The stem root was quite long and had excessive root hairs after 9 days, making it difficult to measure RC in an Eppendorf tube. Therefore the cold-treatment period was determined to be 9 days. The difference in RC between cold-sensitive and cold-tolerant plants was apparent for 1 h cold treatment (Fig. 2A). The RC for cold-tolerant seedlings increased to 8.1 at 24 h, but at 48 h the RC for DX decreased to 4.4 whereas that for NJ increased to 12.9. In addition, NJ had high RC of 16.2 at 72 h whereas the cold-tolerant DX had low RC of 5.3. The high RC in NJ can be explained by the observation that more intracellular liquids leak into the environment per unit time (Fig. 2B).

**Table 1:** RC of parents at different growth stages.

	Days of growth							
	4	5	6	7	8	9	10	11
DX	3.6±1.8	4.2±0.9	4.8±1.9	4.4±1.6	4.4±1.6**	4.7±2.2**	4.4±1.8**	4.8±1.6**
NJ	4.4±0.8	4.7±1.2	6.2±0.8	7.9±2.3	10.1±2.4**	11.9±1.2**	11.7±2.5**	12.7±1.9**

**Significantly different between two parents at *P* = 0.01.

### QTLs for Cold Tolerance

**Figure 3:**
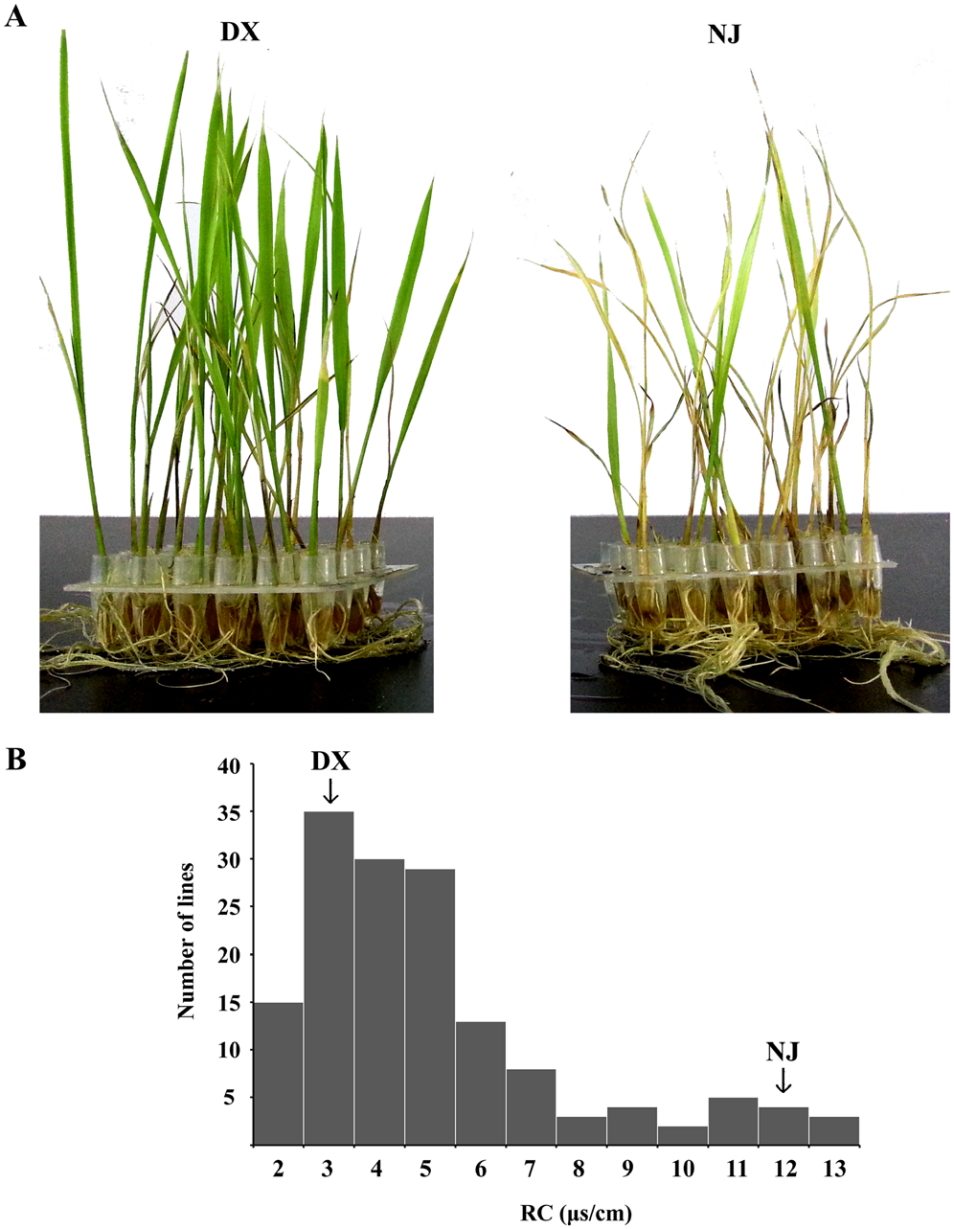
Cold-induced injury phenotype of parents and frequency distribution of BC_2_F_1_ plants after cold treatment. A. Cold-induced injury phenotype of DX and NJ after exposure to 4°C for 48 h. B. Frequency distribution of the RC values among BC_2_F_1_ population. Arrows indicate the mean scores of DX and NJ.

After recovery at 28°C, DX grew normally, whereas NJ showed cold-induced injuries (Fig. 3A). The RC was 2.9 for the cold-tolerant DX and 12.1 for NJ, and the distribution of the RC phenotypes among the BC_2_F_1_ population was continuous and skewed towards tolerance (Fig. 3B).

**Figure 4:**
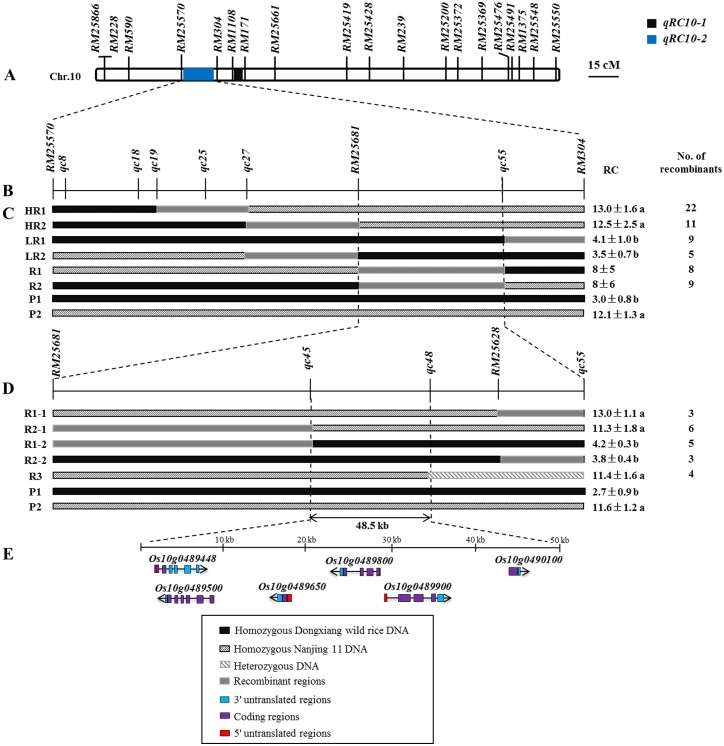
Genetic and physical map covering *qRC10-2*. A. Location of *qRC10-2* on rice chromosome 10. B. The linkage map shows six indels and a SSR marker. C. Progeny test of homozygous recombinants delimited *qRC10-2* to the region between the markers *RM25570* and *RM304*. The number of recombinants in each group and the phenotypic difference of each group from controls are shown. D. Further fine mapping of *qRC10-2*. E. Six genes in the candidate region. ^a^Significantly different from P1 at *P* = 0.01; ^b^significantly different from P2 at *P* = 0.01. P1: DX; P2: NJ.

A total of 653 SSR markers were used to screen polymorphisms between the two parents. Only 113 markers were codominant, with the polymorphism of only 17.3%; all the 113 developed markers were mapped essentially and uniformly to the 12 chromosomes. Based on this, two seedling cold-tolerant QTLs, named *qRC10-1* and *qRC10-2* (Fig. 4A), were detected on chromosome 10 via composite interval mapping. *qRC10-1* (LOD = 3.1) was mapped at 148.3 cM between *RM171* and *RM1108*, and *qRC10-2* (LOD = 6.1) was mapped at 163.3 cM between *RM25570* and *RM304*, which accounted for 9.4% and 32.1% of phenotypic variances, respectively (Table 2).

**Table 2:** QTLs for RC detected in the BC_2_F_1_ BILs at seedling stage.

Trait	Chromosome	Marker interval	Position (cM)	LOD	PVE	Additive effect
*qRC10-1*	10	*RM171*–*RM1108*	148.3	3.1	9.4	2
*qRC10-2*	10	*RM25570–RM304*	163.3	6.1	32.1	4.3

LOD: logarithm of odds; PVE: percentage of total phenotypic variance explained by the QTL;

The LOD threshold for detecting QTLs by composite interval mapping was 2.5.

### Fine Genetic and Physical Mapping of *qRC10-2*

To obtain a fine map of *qRC10-2*, 45 molecular markers were developed between *RM25570* and *RM304*. As shown in Table S3, 10 of these markers showed codominant polymorphisms between DX and NJ, consisting of 2 SSR markers (*RM25681* and *RM25628*) and 8 indel markers (*qc8*, *qc18*, *qc19*, *qc25*, *qc27*, *qc45*, *qc48*, and *qc55*). The arrangement of these markers is shown in Fig. 4B and 4D.

Using the markers *RM25570* and *RM304*, a total of 211 BC_5_F_2_ BIL recombinants were identified. Among them, 64 showed the homozygous genotype for DX or NJ alleles and were divided into six groups (LR1, LR2, HR1, HR2, R1 and R2) based on the genotypes of *qc8*, *qc18*, *qc19*, *qc25*, *qc27*, *RM25681* and *qc55* (Fig. 4C). Group LR1 (RC = 4.1) had DX alleles to the left of *qc55*, and group LR2 (RC = 3.5) had DX alleles to the right of *RM25681*, indicating that *qRC10-2* was located upstream of *qc55*. Groups R1 and R2 had recombinant loci between *RM25681* and *qc55*, and there was a large range in RC for these groups. Based on this, we deduced that *qRC10-2* was located between *RM25681* and *qc55* (Fig. 4C).

Based on the genotypes of the three polymorphic markers *qc45*, *qc48* and *RM25628* (Fig. 4D), groups R1 and R2 were further divided into four subgroups: R1-1, R2-1, R1-2 and R2-2. Then, according to the RC data, the candidate region was narrowed to 68.7 kb between *qc45* and *RM25628*, and *qc45*, *qc48* and *RM25628* were used to rescreen all mapped plants. Finally, we obtained four BC_5_F_2_ plants with high RC of 11.4 (named group R3) heterozygous in the *qc48* allele. Therefore, *qRC10-2* between *qc45* and *qc48* was restricted to 48.5 kb based on the Nipponbare sequence, in which a bacterial artificial chromosome clone (AC146481.2) contained six candidate genes, as shown in Fig. 4D and 4E.

### Effects of *qRC10-2* on Cold Tolerance at the Seedling and Maturity Stages

**Figure 5:**
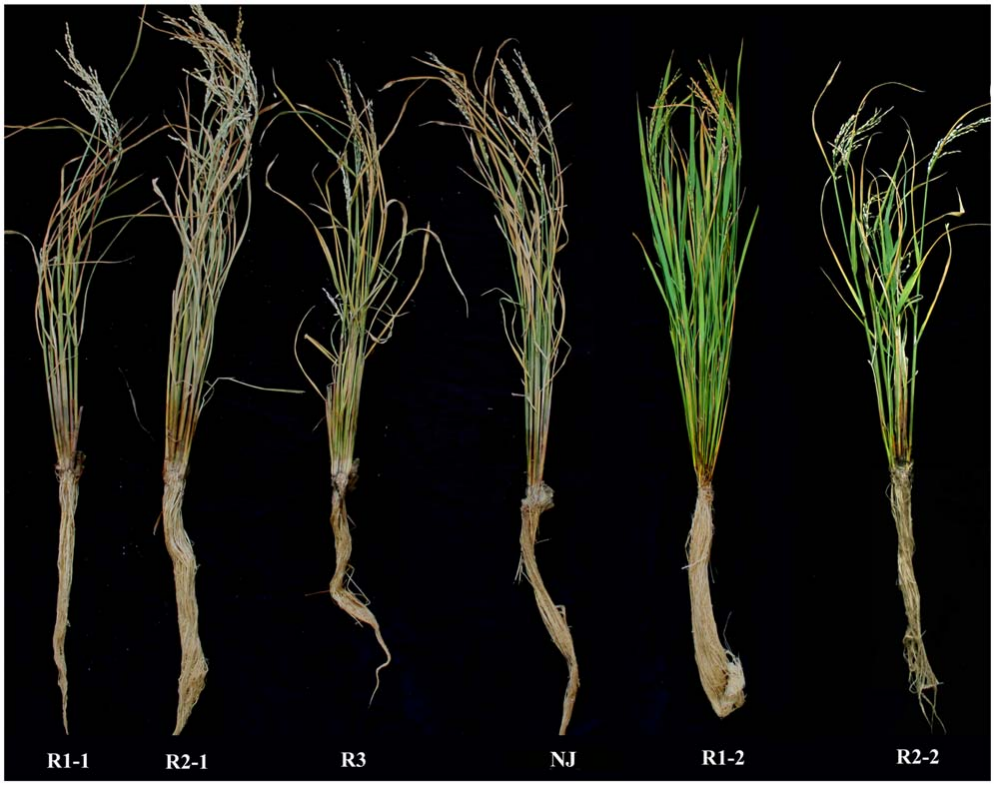
Cold-induced phenotype of the BC_5_F_3_ lines containing or lacking *qRC10*-*2* after heading at the 15-day stage.

Subgroups R1-1, R2-1, R1-2 and R2-2 were used to determine PSRs. The PSRs for R1-1 and R2-1 with high RC values were 25–44% in seedlings and 21–35% in mature plants, whereas those for the subgroups R1-2 and R2-2 with low RC values were 52–60% in seedlings and 48–57% in mature plants. The correlation coefficients between RC and the PSR at the seedling and maturity stages were –0.85 and –0.9 (*P* = 0.01), respectively (Table 3), indicating that a lower RC value was correlated with a higher PSR. Lines with *qRC10*-*2* grew normally after 24 h under 5±1°C treatment, and lines lacking *qRC10*-*2* showed necrosis or death at the maturity stage (Fig. 5). These results indicated that *qRC10*-*2* contributed to rice cold tolerance at both stages.

**Table 3:** Correlations between RC and PSR of recombinants at seedling and maturity stages.

Group	R1-1		R2-1						R1-2					R2-2					Correlation coefficient
Name of recombinant	L4	L114	L21	L56	L77	L92	L104	L147	L32	L44	L96	L123	L137	L48	L81	L156	DX	NJ	
RC (µs/cm)	12.3±0.9	11.9±1.4	10±4.5	12.9±1.9	14.2±2.8	13.8±0.8	16.2±6.9	12.6±3.4	6.7±1.6	8.7±1.7	6.2±1.9	5.4±0.5	5.6±1.6	4.8±2.0	5.1±1.8	4.7±2.7	3.1±0.6	13.9±1.2	/
PSR of seedling stage (%)	30±14^a^	27±6^a^	29±12_a_	25±11^a^	44±8^a^	26±9^a^	32±14^a^	41±14^a^	56±8^b^	52±6^b^	55±12^b^	56±10^b^	53±9^b^	60±10^b^	59±9^b^	56±12^b^	78±10^b^	34±13^a^	–0.85**
PSR of maturity stage (%)	21±12^a^	31±16^a^	34±9^a^	26±7^a^	22±15^a^	35±7a	23±11^a^	33±10^a^	57±8^b^	54±6^b^	49±6^b^	54±6^b^	48±7^b^	52±12^b^	56±8^b^	52±9^b^	66±6^b^	25±6^a^	–0.9**

**P = 0.01.

a Significantly different from DX at *P* = 0.01;

b Significantly different from NJ at *P* = 0.01;

RC: root conductivity; PSR: plant survival rate.

### Identification of Cold-inducible Genes in the Candidate Region

*O. rufipogon* Griff., common wild rice, is the ancestor of cultivated rice [40]–[42]. The collinearity of cold-tolerant genes between different species and the conservation in the cold-responsive pathway has been reported [30], [43], [44]. Therefore, Nipponbare (*japonica*) was selected as the reference genome to screen candidate genes. Table 4 lists the predicted functions of the six candidate genes in IRGSP1.0 (http://rapdb.dna.affrc.go.jp/).

**Table 4:** Candidate genes in the *qRC10*-*2* region.

ORF	Putative protein function	Physical location	AA	Gene size (bp)	BAC ID
Os10g0489448	Unknown	18,572,698–18,575,727	125	1425	AC146481.2
Os10g0489500	Terpene synthase	18,572,698–18,575,857	512	1544	AC146481.2
Os10g0489650	Unknown	18,579,905–18,580,739	101	835	AC146481.2
Os10g0489800	Unknown	18,588,277–18,590,938	384	1363	AC146481.2
Os10g0489900	Putative DNA replication initiation protein	18,591,175–18,595,632	574	2524	AC146481.2
Os10g0490100	Pectin lyase fold family protein	18,598,558–18,599,387	214	830	AC146481.2

ORF: open reading frame; BAC: bacterial artificial chromosome.

**Figure 6:**
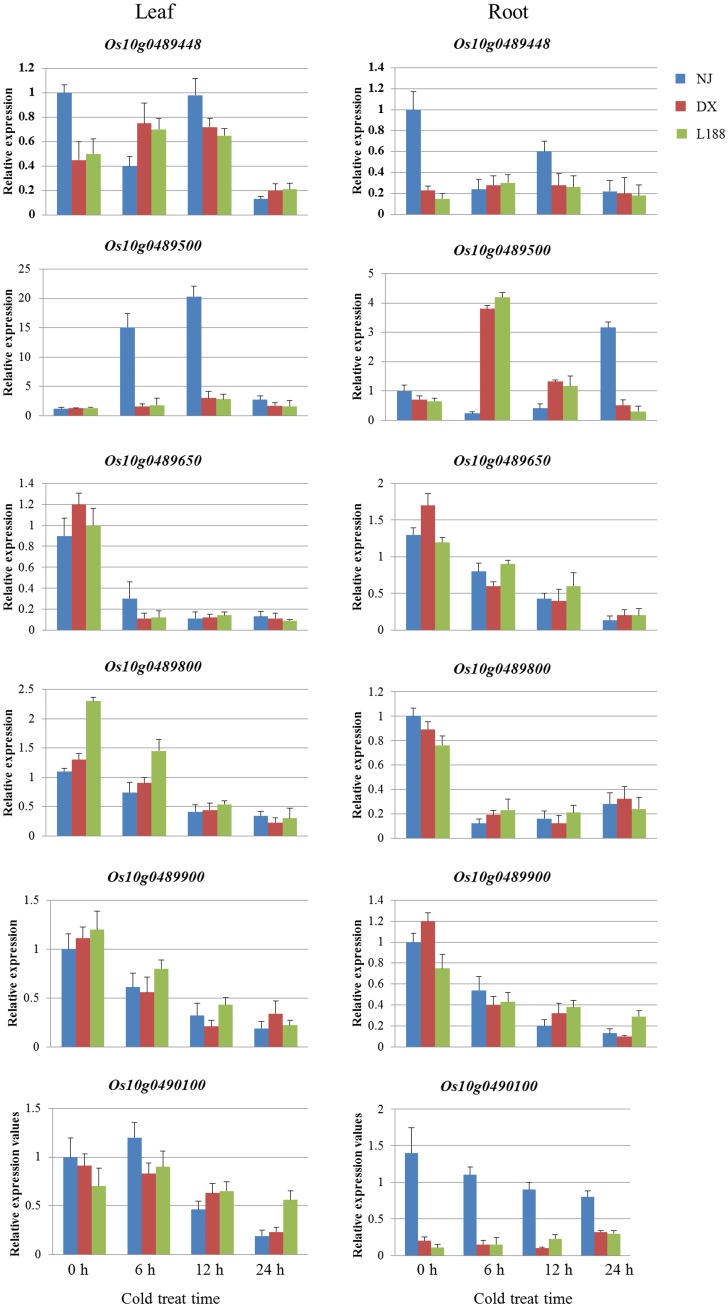
Expression of the candidate genes for plants subjected to 4°C cold treatment for 0, 6, 12, or 24 h as assessed by real-time PCR.

To further identify genes related to cold tolerance, real-time PCR was used to assess gene expression in the leaf and root tissues after 0, 6, 12, and 24 h of cold treatment (Fig. 6). The results suggested that *Os10g0489448*, *Os10g0489650*, *Os10g0489800*, and *Os10g0489900* shared a similar gene expression profile in DX, L188 and NJ. *Os10g0489500* was induced under cold stress and showed high levels of inducible expression in the leaves of NJ at 6 h and 12 h, whereas no significant variation was observed in DX. This pattern was different from that in the roots, as it was highly expressed in the roots of DX at 6 h but not up-regulated in NJ until 24 h. *Os10g0490100* in the roots of NJ showed higher expression than in DX before and after cold treatment. Therefore, *Os10g0489500* and *Os10g0490100* were considered as the genes associated with cold tolerance in the *qRC10*-*2* region.

## Discussion

Cold stress is one of the major environmental concerns in rice cultivation, especially in low-temperature and high-altitude areas [45]. The rice subspecies *indica* and *japonica* showed cold injury symptoms below 18°C and 15°C, respectively [46]. Under low-temperature stress, the root cell plasma membrane might become damaged and less fluid, resulting in functionality loss, which in turn caused increased cell membrane permeability and intracellular material’s extravasation into the external environment [47], [48], [49]. In our present study, RC was used as a cold phenotype indicator reflecting the integrity and permeability of root membrane at low temperatures. With prolonged treatment time, the RC values of DX and NJ increased gradually, indicating cumulative cold injuries in both parents. Besides, more serious damages accumulated in the NJ root membrane. During the recovery period, high-RC plants showed wilting leaves and retarded growth, but low-RC plants maintained green leaves. Such a cold-tolerant phenotype had a consistent effect on plants at the maturity stage with *qRC10*-*2*. Moreover, the correlation coefficients (–0.85 and –0.9) between RC and the PSR were significant in seedlings and mature plants (*P = 0.01*), indicating that RC could be used as a reliable index for evaluating cold tolerance in rice.

In previous studies, most cold-tolerant QTLs at the seedling stage were detected using recombinant inbred lines derived from *indica/japonica* cultivars [12], [18], [50], [51]. However, QTL analysis on a wider range of variations from wild species could be equally important. Koseki et al. identified the cold-tolerant QTL *qCtss10* (LOD = 3.0) from W1943 (*O. rufipogon*) on chromosome 10, which accounted for 9.8% of phenotypic variations [17]. In our study, two QTLs *qRC10*-*1* and *qRC10*-*2* were identified on the same chromosome in DX. A QTL for cold tolerance (*qCTSS-10*) was also identified in seedlings by Yang et al. [52]. *qCTSS-10* for 7°C cold treatment was mapped by high-throughput sequencing in the F_3_ mapping population from a cross between a cold-tolerant *japonica* rice variety (Nipponbare) and a cold-sensitive *indica* variety. The interval of *qCTSS-10* (15.48–21.06 Mb) overlapped that of *qRC10*-*1* and *qRC10*-*2*, indicating that these QTLs may be controlled by the same cold-tolerant gene. Liu et al. also identified the same QTL (*qCST10*, LOD = 2.4) with 12% of the phenotypic contribution from the cold-tolerant line IL112 [53]; all early seedlings of the line IL112 survived normally for 9 days at 4–5°C. A molecular marker tightly linked to *qCST10* was *OSR33*, which was the same marker as *RM171* linked to *qRC10*-*1* and *qRC10*-*2* according to the Gramene marker data (http://www.gramene.org/). As described above, the same cold-tolerant QTL was identified on chromosome 10 among different parents and cold-treatment conditions based on a different cold tolerance index, suggesting that *qRC10*-*2* was a stable and reliable cold-tolerant QTL for the rice seedling stage. Further studies on the candidate gene(s) in this region will aid in elucidating the molecular mechanism underlying rice cold tolerance.

In the candidate region, *Os10g0489500* and *Os10g0490100* were differentially expressed between cold-tolerant and -sensitive plants. *Os10g0489500* encoded a terpene synthase that was localized to the cytoplasm. In our study, *Os10g0489500* was up-regulated in response to cold stress in the leaves of NJ. In the roots, however, its expression in DX and L188 was ∼18-fold higher than that in NJ, suggesting that the root may be a key organ for the *Os10g0489500* activity. Yang et al. also identified *Os10g0489500* as a gene up-regulated by low temperatures in a cold-tolerant variety [52]. After analyzing differentially-expressed transcripts in *indica* (K354) and *japonica* (C418) under controlled conditions (25°C) and cold stress (4°C) for 48 h, Zhang et al. identified two terpene synthase genes (*Os02g36140* and *Os08g07100*) that were associated with signal transduction cascades from K354 during cold-stress responses in seedlings [44]. Another gene differentially-expressed between cold-tolerant and -sensitive plants was *Os10g0490100*, which encoded virulence factors that degraded the pectin components of the plant cell wall [54], [55]. Pectin functioned as an adhesive in cell wall formation, and the pectin content directly influenced cell wall integrity, which was related to rice cold tolerance as reported previously [56]–[58]. Solecka et al. found that cold acclimation induced an increase in the pectin content in the cell wall and increased tolerance to freezing [59]. A mutation in *QUASIMODO1*, a gene encoding the glycosyl transferase required for pectin synthesis, caused reduced pectin content, leading to less cell adhesion and faster water loss during cold stress in common rice plants compared with the wild type [58]. Using Zhonghua 10 plants, Liu et al. overexpressed OsBURP16, a polygalacturonase 1β that degraded pectin, and showed that an increase in the polygalacturonase activity led to a decrease in the pectin content in the cell wall, resulting in more severely curled leaves in the transgenic plants [53]. When the transgenic seedlings were exposed to 4°C for 72 h and allowed to recover for 7 days under normal conditions, the PSR of Zhonghua 10 plants was 42%, and that of the transgenic plants OE3 and OE4 was only 28% and 4%, respectively [60]. The above studies suggested that pectin degradation in the cell wall affected cell wall integrity and decreased tolerance to cold stress. In our present study, *Os10g04901*00, a pectin lyase fold family protein that participated in pectin degradation, was more highly expressed in NJ roots than in DX and L188 roots before and after cold treatment, indicating that fewer pectin was maintained in cold-sensitive plants.

In conclusion, our data provided useful information for cultivating cold-tolerant rice varieties by pyramiding *qRC10*-*2* based on other QTLs and also offered a molecular basis for rice cold tolerance mechanisms at the seedling and maturity stages.

## Supporting Information

Figure S1 **Genetic background recovery rates determined among the BC_4_F_2_ plants.**
(JPG)

Table S1 Primer sequences for SSR markers.
(DOCX)

Table S2 Real-time PCR primer sequences for candidate genes.
(DOCX)

Table S3 Primer sequences and sizes of polymorphic markers developed for fine mapping locus *qRC10-2*.
(DOCX)
