# Supplementary figures and images for: Fine Mapping of qRC10-2, a Quantitative Trait Locus for Cold Tolerance of Rice Roots at Seedling and Mature Stages
Source: PLoS One. 2014 May 1;9(5):e96046. doi: 10.1371/journal.pone.0096046 (PMC4006884; doi:10.1371/journal.pone.0096046)

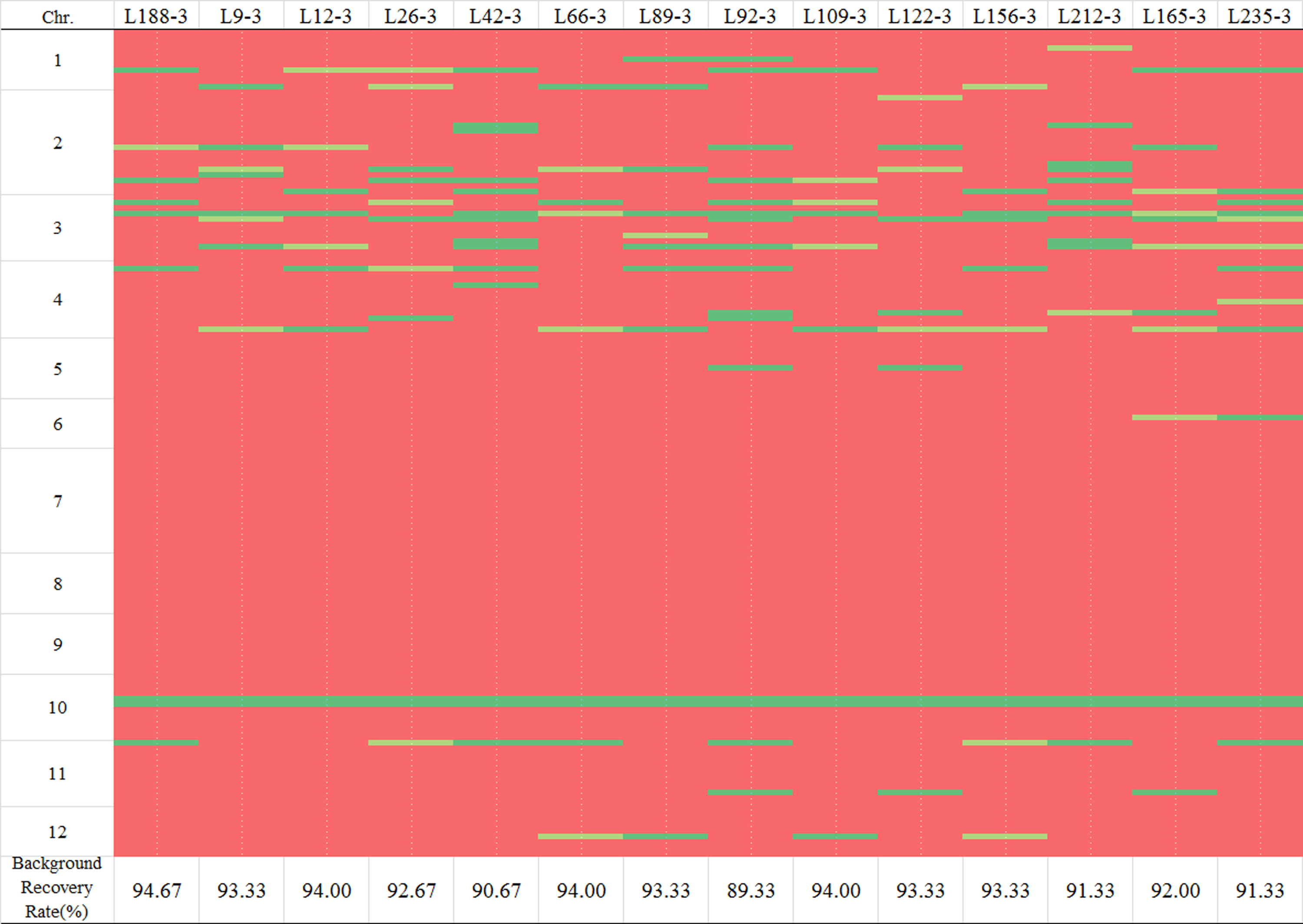

Supplement: Figure S1 — Genetic background recovery rates determined among the BC4F2 plants. (JPG) [file pone.0096046.s001.jpg]
