# Supplementary material for: Fine Mapping of qRC10-2, a Quantitative Trait Locus for Cold Tolerance of Rice Roots at Seedling and Mature Stages
Source: PLoS One. 2014 May 1;9(5):e96046. doi: 10.1371/journal.pone.0096046 (PMC4006884; doi:10.1371/journal.pone.0096046)
Supplement: Table S1 — Primer sequences for SSR markers. (DOCX) [file pone.0096046.s002.docx]

| **Marker** | **Chr.** | **Forward** | **Reverse** |
| --- | --- | --- | --- |
| RM11635 | 1 | TTGTCACCCTTACTAGGATCAGC | GTGTGACTCTTGATGTAACTCAGC |
| RM11650 | 1 | TCCACGGTGCAATAAATCACTCC | GGCATTTCTCAATTGCTGGTTCG |
| RM1254 | 1 | GCCCTGTGTATTCATGCATG | CATGCAGATTAGAGGTGGAC |
| RM129 | 1 | TCTCTCCGGAGCCAAGGCGAGG | CGAGCCACGACGCGATGTACCC |
| RM1297 | 1 | GTGCCTTACAACTCAACGAC | CACTCCCAGTTCAGTACGTC |
| RM283 | 1 | GTCTACATGTACCCTTGTTGGG | CGGCATGAGAGTCTGTGATG |
| RM3482 | 1 | TTGTTGTCAAGCTACGGTGG | CTGCTTCGTGATGTTGTTGG |
| RM5853 | 1 | GTAGCAGTAGTAGTACCACCGACG | ACCAGTGTGGTTTCTTTGGG |
| RM6289 | 1 | GGGTTTTGCATTCTTTGGGG | CTTAGCTACAACCTTCGCCC |
| RM6521 | 1 | GAGGCAGTGGGACATTGC | CGAGGAGAATACGCAAAACG |
| RM8004 | 1 | TTGACCAAAGGTGATTGTAAT | CTTGATGAGTTTCATGAGCA |
| RM106 | 2 | CGTCTTCATCATCGTCGCCCCG | GGCCCATCCCGTCGTGGATCTC |
| RM12380 | 2 | GCTCCCAACTAGTGCAAGTACACG | CATGGCTAACTAGATCGACACACG |
| RM12585 | 2 | GTGAACCATCACTTGTTGAACC | GCGACACATATAGAGAGTTTGTCC |
| RM13296 | 2 | GGGTGGTGTAACGGAGAGATAACG | CTCTTCCCTCCTTCCTTCATGC |
| RM13922 | 2 | CGGCATTGTGGCGCTGTAGG | GCATCTGGCAAAGCCAATTTCG |
| RM3763 | 2 | TCTCTGAACACACCCACACC | TGTTTTGATCTCAGCTCCCC |
| RM424 | 2 | TTTGTGGCTCACCAGTTGAG | TGGCGCATTCATGTCATC |
| RM425 | 2 | CCAACGAAGATTCGAAGCTC | CAGCACCATGAAGTCGCC |
| RM425-2 | 2 | CCAACGAAGATTCGAAGCTC | CAGCACCATGAAGTCGCC |
| RM485 | 2 | CACACTTTCCAGTCCTCTCC | CATCTTCCTCTCTTCGGCAC |
| RM5210 | 2 | CTAAATGACAAAAGATAGCA | AAATTCTGACTTGTATGACA |
| RM526 | 2 | CCCAAGCAATACGTCCCTAG | ACCTGGTCATGACAAGGAGG |
| RM550 | 2 | CTGAGCTCTGGTCCGAAGTC | GGTGGTGGAAGAACAGGAAG |
| RM5614 | 2 | GTTGCAGGACAGCAGGAACAGC | CACTCCACCATTCCACCAACTAGC |
| RM6023 | 2 | TGAGTGTTTGCGGGAGGAGAGC | CAGGGTGAGAGGTTCCAGTCAGG |
| RM6233 | 2 | AGAACGGCGACACGTGGAC | ACGATGACGCCCATGATG |
| RM6367 | 2 | CAGACAGAACAGCGGTCAAG | GATGGATGGATGGATTGGAG |
| RM6509 | 2 | GGTGTTTTGTGGTGTTGTGC | CTCGAACTGCGAGTAGGACC |
| RM6895 | 2 | TCAAATAAGATGGACGGTC | CGCCACCACAGTAGTACTAG |
| RM1164 | 3 | CGTTTCTCCGAGAAAAGTCG | CAAGGTGGTCGTTGAGGC |
| RM15280 | 3 | AGTTGAAGTGTTGACCGCAATCC | GTAGGTAGAGGCTCTGGCAGTCG |
| RM2334 | 3 | CATGCATCTGATCTGATTAT | TGTGAAGAGTACAAGTAGGG |
| RM3436 | 3 | GCATCCCGGTGACTAGTACG | TGTGCATGTGGTAAGGAACC |
| RM3646 | 3 | ACTAGAGCACCCTCGCTGAG | CTCAGCCACCCCATCAAC |
| RM3716 | 3 | GTCGTTCGGTTGACTCGTTG | CACACATATATACCCCCCCC |
| RM569 | 3 | GACATTCTCGCTTGCTCCTC | TGTCCCCTCTAAAACCCTCC |
| RM571 | 3 | GGAGGTGAAAGCGAATCATG | CCTGCTGCTCTTTCATCAGC |
| RM5864 | 3 | ATTAGTACCGTGTGGTCCGC | GACCGAATTGGTGATCGATC |
| RM7117 | 3 | AGTTGGCTGGTTGCTACCAC | AGGGTTCCCTGGCTACTCAC |
| RM7134 | 3 | TTCTACGCGTGCATATGGTC | ATGGCGCCATTAGGTTAGAG |
| RM1155 | 4 | AGGGAGTGTGGCAACTATGC | GGGAGGAGTGAGAAGGGATC |
| RM142 | 4 | CTCGCTATCGCCATCGCCATCG | TCGAGCCATCGCTGGATGGAGG |
| RM17102 | 4 | AACGTACTTCTGCCTCCCACAGG | GTCCCTTTGCAGTGTACATGACG |
| RM185 | 4 | AGTTGTTGGGAGGGAGAAAGGCC | AGGAGGCGACGGCGATGTCCTC |
| RM273 | 4 | GAAGCCGTCGTGAAGTTACC | GTTTCCTACCTGATCGCGAC |
| RM3317 | 4 | CCTGACAGAAGAATGGTACACC | TGTGGCTTCTCGTTGAGTTG |
| RM335 | 4 | GTACACACCCACATCGAGAAG | GCTCTATGCGAGTATCCATGG |
| RM349 | 4 | TTGCCATTCGCGTGGAGGCG | GTCCATCATCCCTATGGTCG |
| RM349-2 | 4 | TTGCCATTCGCGTGGAGGCG | GTCCATCATCCCTATGGTCG |
| RM3839 | 4 | AATGGGACCAGAAAGCACAC | AAAAAGAGCATGGGGGCTAC |
| RM6314 | 4 | GATTCGTGTCGGTTGTCAAG | GGTTCAGGGACGAATTTCAG |
| RM6629 | 4 | TAAACGGTGTGCAGCTTCTG | TATTATGGGCGGTCGCTAAC |
| RM6659 | 4 | GTTGTTGTTGTTGTGGACGG | CTGCCCTGAGTCCTATGAGG |
| RM1024 | 5 | GCATATACCATGGGGATTGG | GGGATTGGGATAATGGTGTG |
| RM17886 | 5 | AAGAGATGTGTGGTTCAACTCC | CCTAGGTATTCCCAAGAGAGTCC |
| RM18326 | 5 | AACGGATGGATGGATGCTCACC | CTTGCACCATAGTCGCAATCACC |
| RM18457 | 5 | ATCCTCCACCGCTCAAGAACACG | CGAGGCCATTCATCGAACAAAGC |
| RM267 | 5 | TGCAGACATAGAGAAGGAAGTG | AGCAACAGCACAACTTGATG |
| RM274 | 5 | CCTCGCTTATGAGAGCTTCG | CTTCTCCATCACTCCCATGG |
| RM3664 | 5 | CGCCTGCAAAAAAGGTAGAG | GATCAAAGGAACCCCCGTAG |
| RM3853 | 5 | AACATATGCTATGTGCCCTT | GGAGTTATCAGCAAATGCTC |
| RM5140 | 5 | GACGAGGTTGTTTATTAGTG | CTTATTTTCACGTGTACGTT |
| RM5907 | 5 | TGCTGTCTCCACTTCCCTTC | AAGGAGGCGTGCTTAACAGG |
| RM6082 | 5 | AACCCTAGAATCGGCGCTG | CACCGATGACAACGAGGAC |
| RM162 | 6 | GCCAGCAAAACCAGGGATCCGG | CAAGGTCTTGTGCGGCTTGCGG |
| RM19558 | 6 | CATCTGATCTCGCATGCACTTGG | GTCTCTCTGCCGCTGGATCG |
| RM2523 | 6 | CCGTAGGTCTTCAAGTGATA | GCAAAACCGAACTAAAATTA |
| RM510 | 6 | AACCGGATTAGTTTCTCGCC | TGAGGACGACGAGCAGATTC |
| RM540 | 6 | GCCTTCTGGCTCATTTATGC | CTAGGCCTGCCAGATTGAAC |
| RM5463 | 6 | ACCCTTGCAGACAACGTACC | ATATACCAGCAGCTGCATGC |
| RM587 | 6 | ACGCGAACAAATTAACAGCC | CTTTGCTACCAGTAGATCCAGC |
| RM588 | 6 | GTTGCTCTGCCTCACTCTTG | AACGAGCCAACGAAGCAG |
| RM7551 | 6 | TCACCTCCTTCTGCCATCTC | CTAACTCACCTCCAGCCTGC |
| RM1093 | 7 | AGGTTGATGAACCCGATGAG | CTAGCTGCAGAACGGAGGAG |
| RM11 | 7 | TCTCCTCTTCCCCCGATC | ATAGCGGGCGAGGCTTAG |
| RM1373 | 7 | ATGAGGTTCAAAATGAGACG | TTAAGCTACTGTCTGCCTCC |
| RM172 | 7 | TGCAGCTGCGCCACAGCCATAG | CAACCACGACACCGCCGTGTTG |
| RM172-2 | 7 | TGCAGCTGCGCCACAGCCATAG | CAACCACGACACCGCCGTGTTG |
| RM180 | 7 | CTACATCGGCTTAGGTGTAGCAACACG | ACTTGCTCTACTTGTGGTGAGGGACTG |
| RM20897 | 7 | TTTACACACATGCTCCTTCTGC | AAAGCAACCACCTCCATTATCC |
| RM21160 | 7 | AAACAAATCACCCTCGTGGATCG | TGGGAACACCACAATGTGAAGC |
| RM21911 | 7 | ACTTGGGAAATATGGTGCGAACG | CCTGAAGTTCAACACCAGATCAATGC |
| RM3224 | 7 | AGACGTACACCCCGAACTTG | GAGGTGTTCGGAGTGAGGAG |
| RM336 | 7 | CTTACAGAGAAACGGCATCG | GCTGGTTTGTTTCAGGTTCG |
| RM3394 | 7 | CCCTTACGTGCAGTACATTG | ATGCAGGCTACTTACTAGCG |
| RM3859 | 7 | TTGCAGATCGGTTTCCACTG | GGTCCTGGATTCATGGTGTC |
| RM420 | 7 | GGACAGAATGTGAAGACAGTCG | ACTAATCCACCAACGCATCC |
| RM432 | 7 | TTCTGTCTCACGCTGGATTG | AGCTGCGTACGTGATGAATG |
| RM533 | 7 | GCAACTGCTCTACGCCTCTC | CCTGAGGCTTCACCTACTCG |
| RM542 | 7 | TGAATCAAGCCCCTCACTAC | CTGCAACGAGTAAGGCAGAG |
| RM542-2 | 7 | TGAATCAAGCCCCTCACTAC | CTGCAACGAGTAAGGCAGAG |
| RM5543 | 7 | ACCACTTGCTGGAATCCTTG | GCAAATTCTGGGCTATCTGC |
| RM6776 | 7 | AGCCCGGACATGCAAAAC | GAAGCAGGCGAAATCTCCTC |
| RM223 | 8 | GAGTGAGCTTGGGCTGAAAC | GAAGGCAAGTCTTGGCACTG |
| RM25 | 8 | GGAAAGAATGATCTTTTCATGG | CTACCATCAAAACCAATGTTC |
| RM256 | 8 | GACAGGGAGTGATTGAAGGC | GTTGATTTCGCCAAGGGC |
| RM337 | 8 | GTAGGAAAGGAAGGGCAGAG | CGATAGATAGCTAGATGTGGCC |
| RM339 | 8 | GTAATCGATGCTGTGGGAAG | GAGTCATGTGATAGCCGATATG |
| RM3845 | 8 | AGCTCGATCTCCTCTCTAGACC | GCTTCAGCCTTCAGGTCAAC |
| RM408 | 8 | CAACGAGCTAACTTCCGTCC | ACTGCTACTTGGGTAGCTGACC |
| RM419 | 8 | TCTCCTTTGGTATGCGTGTG | GCTGCTGCTCCACTTTTCTC |
| RM44 | 8 | ACGGGCAATCCGAACAACC | TCGGGAAAACCTACCCTACC |
| RM556 | 8 | ACTCCAAACCTCACTGCACC | TAGCACACTGAACAGCTGGC |
| RM7631 | 8 | GGTCACTCATGGTGCATGTC | CACACTCACTCACTCACTTGAC |
| RM107 | 9 | AGATCGAAGCATCGCGCCCGAG | ACTGCGTCCTCTGGGTTCCCGG |
| RM205 | 9 | CTGGTTCTGTATGGGAGCAG | CTGGCCCTTCACGTTTCAGTG |
| RM205-2 | 9 | CTGGTTCTGTATGGGAGCAG | CTGGCCCTTCACGTTTCAGTG |
| RM215 | 9 | GAGCAGCAAGAGCAGCAGAGG | CATGCTCGACTTCAGAAGCTTGG |
| RM24122 | 9 | GCGGTATGAGTGCGTTTATAGGG | CCTAGTTTACGGATCTGGACATGC |
| RM3164 | 9 | TCCTCCTGCTAGCTGCCTAG | TCGCCTTCCTTTTCACTCAC |
| RM3164-2 | 9 | TCCTCCTGCTAGCTGCCTAG | TCGCCTTCCTTTTCACTCAC |
| RM3165 | 9 | GTTCCGGTCGGGACTAGTTC | GTGGATGGGAGCAGGTGTAC |
| RM6543 | 9 | CGGGCTCCTGAACAGTCTAC | GCAATATCTCATTCTCGGGC |
| RM6543-2 | 9 | CGGGCTCCTGAACAGTCTAC | GCAATATCTCATTCTCGGGC |
| RM7175 | 9 | ACAGTAAACGTGGTGCCTCC | AGAAGTAGCCTCGAGGACCC |
| RM171 | 10 | AACGCGAGGACACGTACTTAC | ACGAGATACGTACGCCTTTG |
| RM25550 | 10 | AAGGAGGAGTACTTAAGGGTAGTGG | TTTATAGGAGCTAAGGTGGAGTGG |
| RM25548 | 10 | AACCAGGAGTAACCAGAGTAACACG | TGTACCATGAGGCCCGTAGG |
| RM1375 | 10 | CTACACGCGCAAACTCTGTC | ATGAAGGTCTAGGCTGCACC |
| RM26491 | 10 | ATTAATTCCACGTCAGGTGTCC | GTAGGGTGGTGTTTGTGTCAGG |
| RM25476 | 10 | CTAACACCGAGCGTGGTGGATGG | GAATCGAATCGGCGGTTTATTCAGG |
| RM25369 | 10 | TAAGATCGTAAGATCGCGGC | AGGCAGGAAGAGGTGGAGG |
| RM25372 | 10 | ATACTCAAACAACCCACACACG | GCTGGTTGAGTTTGTAGGTTACG |
| RM25200 | 10 | GCAATGTTTCTCATGACCTTCG | TGTGGAATATCACGTAGCCAAGC |
| RM25428 | 10 | GCTGAAGAACTTGTTCGCATCC | CGGCGATTACATATCCCTCTTCC |
| RM25419 | 10 | CGGAGACAACGAATGCTCAGTGG | CCAGATCTGCGAGGGAGGATGG |
| RM25661 | 10 | TCTCTACCTGGCGTCCACTAGTTCG | AGGTGGACGTGCTCGATCTGC |
| RM25886 | 10 | GGCTCAATTCGTAGGTGCC | TTCTCCGGTTAACGTGGAAG |
| RM228 | 10 | CTGGCCATTAGTCCTTGG | GCTTGCGGCTCTGCTTAC |
| RM1108 | 10 | GCTCGCGAATCAATCCAC | CTGGATCCTGGACAGACGAG |
| RM239 | 10 | TACAAAATGCTGGGTACCCC | ACATATGGGACCCACCTGTC |
| RM239-2 | 10 | TACAAAATGCTGGGTACCCC | ACATATGGGACCCACCTGTC |
| RM25570 | 10 | ATTAGAGGCACGGGAAGATTGG | CCCACAAAGTTATACGTGGATTGG |
| RM304 | 10 | TCAAACCGGCACATATAAGAC | GATAGGGAGCTGAAGGAGATG |
| RM3773 | 10 | CTGGATGAAAGGATACAACA | CACATTATCTGTCAAGGTCC |
| RM4771 | 10 | ACGTTGATTTCATTCAGGTC | ACGCTAACTGAGAAACATGG |
| RM5348 | 10 | AATCCGATAGGAGTACCGCC | AAGTGTATGGGCTGGAATGG |
| RM590 | 10 | CATCTCCGCTCTCCATGC | GGAGTTGGGGTCTTGTTCG |
| RM1341 | 11 | AACCTGGAGGTGCTGGTCTC | TTTCTCCCCCCCAACCAC |
| RM167 | 11 | GATCCAGCGTGAGGAACACGT | AGTCCGACCACAAGGTGCGTTGTC |
| RM202 | 11 | CAGATTGGAGATGAAGTCCTCC | CCAGCAAGCATGTCAATGTA |
| RM202-2 | 11 | CAGATTGGAGATGAAGTCCTCC | CCAGCAAGCATGTCAATGTA |
| RM26269 | 11 | GGAGGTAGGGAAATCAGGTGAGG | GTGCACGTGACCATAAACACTCC |
| RM27088 | 11 | TGCAAAGTGCAAAGCGTAAAGC | TAGCAGCAGCAGTAGGAACAAGAAGG |
| RM27389 | 11 | ACCGACACCGTCTCCATTATCC | TTGTTTGCCTCCTCTGCAACG |
| RM287 | 11 | TTCCCTGTTAAGAGAGAAATC | GTGTATTTGGTGAAAGCAAC |
| RM3428 | 11 | ATTCATGCTTCCTTTCAGTG | GATTACTGGTTTGCCATTTG |
| RM4601 | 11 | CATACATGTGAACCTGACTG | CTAGCTTAGCATCTCCTCAA |
| RM7277 | 11 | GCTGAACGTTTCAATATGTA | GTTTGTAGGGAGTTTAATGG |
| RM7391 | 11 | GATGCCACATAGCGACTTAG | GTCAATGAGTTCTTCAATTCC |
| SSR4 | 11 | GTTGATGAGCTCCATCGCTC | TCCTCTTAAGCAGCCACCAT |
| RM1036 | 12 | CTCATTTGTCGATTGCCGTC | ATGGGAGGAGTGATCAAACG |
| RM1337 | 12 | GTGCAATGCTGAGGAGTATC | CTGAGAATCTGGAGTGCTTG |
| RM280 | 12 | CATATCCGCATTTGAGTCACTTCG | AACGCCAACTATGGAACTGTTTCG |
| RM28029 | 12 | GGGAAGGAGGAAGGAGGAAAGAGG | TTCACTGGCGGCCACTAACAAGC |
| RM3326 | 12 | CTCATCACCATCGTCACCAC | TCGTCGGGAGAGAGAGAGAG |
| RM5313 | 12 | AATTCCTCCTCTTTCTCCGC | GAGAACATCACGGTGGCC |
| RM5479 | 12 | CTAAGCTCACCATAGCAATC | ATACACTTCTCCCCTCTCTG |
| RM5590 | 12 | TAAGCGATTGAGGTAGCTGG | CCCGTTATAATGAGGGAGGG |
| RM5927 | 12 | TGGTCTCGTCTCTCATGTGC | TAGCCCGGAAGTATGATCCC |
| RM7619 | 12 | CTTGGTATGTATTGGCAGCG | GAGGCAATAGGAGGGGAGAG |
