# Supplementary material for: Fine Mapping of qRC10-2, a Quantitative Trait Locus for Cold Tolerance of Rice Roots at Seedling and Mature Stages
Source: PLoS One. 2014 May 1;9(5):e96046. doi: 10.1371/journal.pone.0096046 (PMC4006884; doi:10.1371/journal.pone.0096046)
Supplement: Table S2 — Real-time PCR primer sequences for candidate genes. (DOCX) [file pone.0096046.s003.docx]

| **ORF** |  | **Primer sequence (5’-3’)** | **Annealing temperature (°C)** | **Product size (bp)** |
| --- | --- | --- | --- | --- |
| *Os10g0489448* | F | ATGTGCTCCTCCGCTTCG | 59 | 159 |
|  | R | CATCCCTCCTGTCATCTCC |  |  |
| *Os10g0489500* | F | ACCACTACTTCCAGGACGAG | 55.5 | 303 |
|  | R | CGATGGCAGATTGAAGGTTT |  |  |
| *Os10g0489650* | F | CGCTGCCCAACGCCGATT | 56 | 149 |
|  | R | ATACCGGCCGCATGCAAA |  |  |
| *Os10g0489800* | F | AGCGTGAGCTTCCACCCAA | 60 | 152 |
|  | R | CCGCCCAAACTACCCTCCTT |  |  |
| *Os10g0489900* | F | TGCTGCTCAGTTGTTGGG | 56 | 258 |
|  | R | ACCTGGGTGGCAATGTTC |  |  |
| *Os10g0490100* | F | AGCGGAGGCGGTGGCTTCTT | 61.5 | 120 |
|  | R | GCAGCACTCGGAGCCGTTGT |  |  |
| *Actin 1* | F | CTTCATAGGAATGGAAGCTGCGGGTA | 56 | 195 |
|  | R | CGACCACCTTGATCTTCATGCTGCTA |  |  |

ORF: open reading frame
