# Supplementary material for: Fine Mapping of qRC10-2, a Quantitative Trait Locus for Cold Tolerance of Rice Roots at Seedling and Mature Stages
Source: PLoS One. 2014 May 1;9(5):e96046. doi: 10.1371/journal.pone.0096046 (PMC4006884; doi:10.1371/journal.pone.0096046)
Supplement: Table S3 — Primer sequences and sizes of polymorphic markers developed for fine mapping locus qRC10-2. (DOCX) [file pone.0096046.s004.docx]

| Primer | Primer sequence (5’-3’) | | Type | Product size (bp) |
| --- | --- | --- | --- | --- |
| *qc8* | F | CAGGAGCCCTAAATGGAGA | Indel | 302 |
|  | R | ACAATGTGGTGCTACAGTAAAAAT |  |  |
| *qc18* | F | CCAAATGCCCAAATCACC | Indel | 203 |
|  | R | CGTCCCAACATCCCCTTCTA |  |  |
| *qc19* | F | AGCCAGAACAGACAGACACTTGA | Indel | 139 |
|  | R | GCTTACGGTTGCTTCAGTTCC |  |  |
| *qc25* | F | CATGGACTAAGGACAATGTCAAGA | Indel | 206 |
|  | R | TGAGTTGTGCAAGCATGAGGTAT |  |  |
| *qc27* | F | GGAGAAGACAACACGGAACG | Indel | 194 |
|  | R | GCTGGGTAGATAAGCAGATGAG |  |  |
| *qc45* | F | CCCATTCCTCCTCGTTCT | Indel | 639 |
|  | R | GGTAGCGGTCCTATCTTGG |  |  |
| *qc48* | F | TCTGATTTGGCATTTGTTAC | Indel | 814 |
|  | R | GTCCACCATAAAGGCACA |  |  |
| *qc55* | F | ATCCTGCTCCTCGTCCCA | Indel | 255 |
|  | R | GAACTAATACCCATCTATCCAACAT |  |  |
| *RM25628* | F | GGTCAAGGCTCAGCCATACTTGG | SSR | 322 |
|  | R | AAGTTGAAGGACGGGTACAATGTCG |  |  |
| *RM25681* | F | AGCCGCTACTTCTACTACCACAGC | SSR | 204 |
|  | R | TCGTCGTCTTCTGGTACATCAGG |  |  |
